# Life-threatening gastrointestinal bleeding during targeted therapy for advanced renal cell carcinoma: a case report

**DOI:** 10.1186/1471-2369-14-141

**Published:** 2013-07-10

**Authors:** Shintaro Fujihara, Hirohito Mori, Hideki Kobara, Takehiro Suenaga, Yuji Hayashida, Mikio Sugimoto, Yoshiyuki Kakehi, Tsutomu Masaki

**Affiliations:** 1 Department of Gastroenterology and Neurology, Kagawa University Faculty of Medicine/Graduate School of Medicine, 1750-1 Ikenobe, Miki-cho, Kita-gun, Kagawa Prefecture 761-0793, Japan; 2 Department of Urology, Kagawa University, Faculty of Medicine, 1750-1 Ikenobe, Miki-cho, Kita-gun, Kagawa Prefecture 761-0793, Japan

**Keywords:** Gastrointestinal bleeding, Temsirolimus, Gastric antral vascular ectasia, Targeted therapy, Argon plasma coagulation

## Abstract

**Background:**

Temsirolimus has important clinical activity in both untreated and previously treated patients with advanced renal cell carcinoma. Targeted therapy–related stomatitis and mucositis have occurred during targeted therapies, but there is no consensus on which strategy is the most effective. We herein report a case in which several sessions of endoscopic hemostasis with argon plasma coagulation (APC) effectively resolved life-threatening gastrointestinal bleeding that had occurred during targeted therapy. This is the first case report of such an adverse drug reaction in the literature.

**Case presentation:**

A 47-year-old female patient with advanced renal cell carcinoma was treated with temsirolimus. Eight weeks after starting targeted therapy, the patient was admitted to our hospital for worsened fatigue, pallor, and hematemesis. A complete blood count showed a marked drop in her hemoglobin level from 10.1 g/dl 4 days earlier to 2.9 g/dl. Esophagogastroduodenoscopy revealed diffuse mucosal bleeding of the antrum. Endoscopy revealed diffuse reddish spots that resembled gastric antral vascular ectasia (GAVE) extending from the pylorus into the antrum. One month after endoscopic hemostasis with APC and stopping temsirolimus, significant improvement was shown in the gastric erythema and GAVE like lesions.

**Conclusion:**

Minor hemorrhagic events are relatively common in patients treated with targeted agents. Life-threatening hemorrhagic events are rarer than minor hemorrhagic complications. In the present case, endoscopic hemostasis with APC effectively prevented severe anemia and blood loss due to gastrointestinal bleeding.

## Background

With the advent of targeted agents for the treatment of renal cell carcinoma (RCC), most patients are being treated continuously for increasingly long periods of time. This has raised new challenges related to management of the associated adverse events [1].

Temsirolimus has important clinical activity in both untreated and previously treated patients with advanced RCC [2]. Severe adverse events associated with temsirolimus are uncommon; however, the most frequent grade 3 or 4 adverse events are anemia, asthenia, and hyperglycemia [3]. Targeted therapy–related stomatitis and mucositis have occurred during targeted therapy, but there is no consensus on which strategy is the most effective.

We herein report a case in which several sessions of endoscopic hemostasis with argon plasma coagulation (APC) effectively resolved life-threatening gastrointestinal bleeding that had occurred during targeted therapy.

## Case presentation

**Figure 1:**
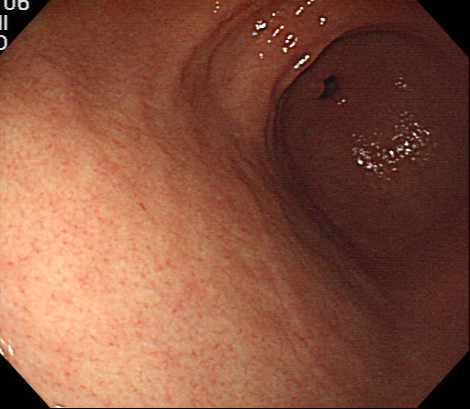
Previous esophagogastroduodenoscopy before starting targeted therapy.

The patient was a 40-year-old woman diagnosed as having left renal cell carcinoma (cT3aN2M1) and adrenal gland metastasis. She was surgically treatment, having radical left nephrectomy in October 2009. There was no history of chronic kidney disease, valvular heart diseases, liver cirrhosis and other disorders. The resected tumor was pathologically diagnosed as collecting duct carcinoma, G3 INFβ, v(+), ly(+) 7 cm pT2. In April 2010, follow-up computerized tomography (CT) revealed newly bronchial lymph node metastasis, and she received initially 30 Gy radiation therapy for lymph node metastasis to prevent bronchial stenosis. After the radiation therapy, she complained of epigastralgia, so we performed esophagogastroduodenoscopy (EGD). However it revealed no abnormality (Figure 1).

Temsirolimus (15 mg/day) was started in June 2012. Her blood test results at various follow-ups showed mild stable anemia (hemoglobin of 10.1–10.7 g/dl), but other laboratory parameters were normal on all occasions, including her chemistry panel and liver function tests.

Eight weeks after starting targeted therapy, the patient was admitted to the Department of Gastroenterology for worsening fatigue, pallor, and hematemesis. A complete blood count showed a marked drop in her hemoglobin level from 10.1 g/dl 4 days earlier to 2.9 g/dl. Her prothrombin time and activated partial thrombin time were normal. She had made no dosage changes to any of her medications, and had not started/stopped any medication during targeted therapy.

**Figure 2:**
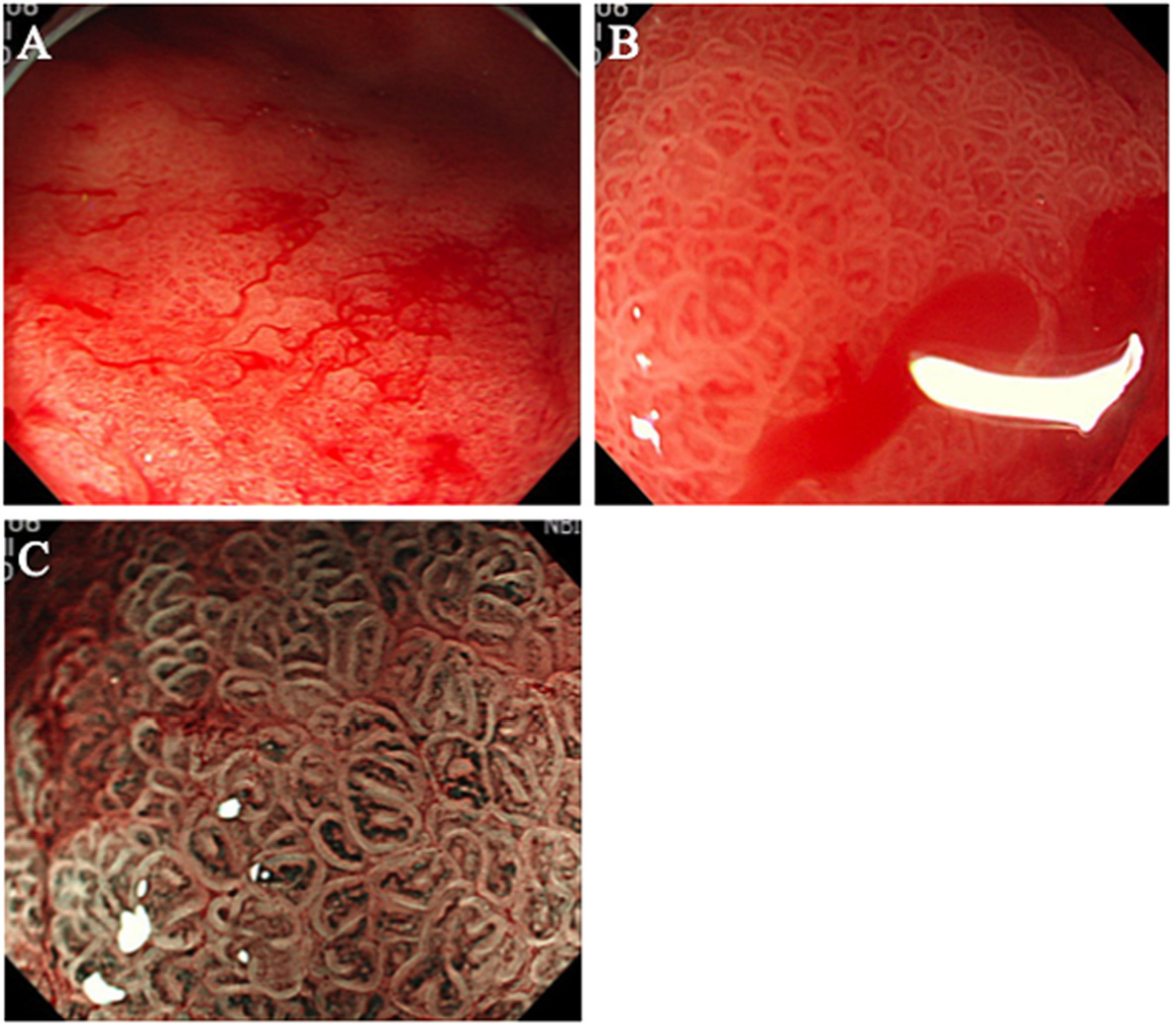
**Esophagogastroduodenoscopy (EGD) when the patient presented with gastrointestinal bleeding. (A)** Esophagogastroduodenoscopy revealed diffuse mucosal bleeding of the antrum that resembled gastric antral vascular ectasia. **(B)** Magnifying endoscopy (ME) findings. The gastric mucosa was friable, and oozing bleeding occurred from ectatic vessel rupture. **(C)** ME with narrow-band imaging revealed congestive and dilated subepithelial capillaries in the background mucosa.

EGD revealed diffuse mucosal bleeding of the antrum (Figure 2A). Endoscopy showed diffuse reddish spots that resembled gastric antral vascular ectasia (GAVE) extending from the pylorus into the antrum. High-resolution magnifying endoscopy (ME) revealed that the mucosa was friable, and oozing bleeding occurred from ectatic vessel rupture (Figure 2B). In the background mucosa, brownish subepithelial capillaries were clearly visualized by ME with narrow-band imaging, and congestive and dilated subepithelial capillaries were seen in the background mucosa (Figure 2C). These findings represented a major change from the EGD performed before targeted therapy. Pathologic examination revealed interstitial fibrosis and extensive edema with capillary and venous dilatation in the submucosa extending into the mucosa.

This complication was most likely an adverse reaction to temsirolimus, because the patient had no underlying medical conditions associated with GAVE and had no evidence of GAVE on EGD before starting temsirolimus.

Treatment with a proton-pump inhibitor was started, and follow-up with repeat endoscopic hemostasis with argon plasma coagulation (APC) was performed once to twice weekly (Additional file 1). The patient required four to six units of packed red blood cells every day, and the need for blood transfusion gradually decreased with the hemostasis treatment. After three sessions of APC, the patient did not need the blood transfusions, and the improvement of GAVE like lesions.

Overall, the patient required a total of four sessions of endoscopic hemostasis and 38 units of transfused blood for severe anemia during these 2 weeks.

**Figure 3:**
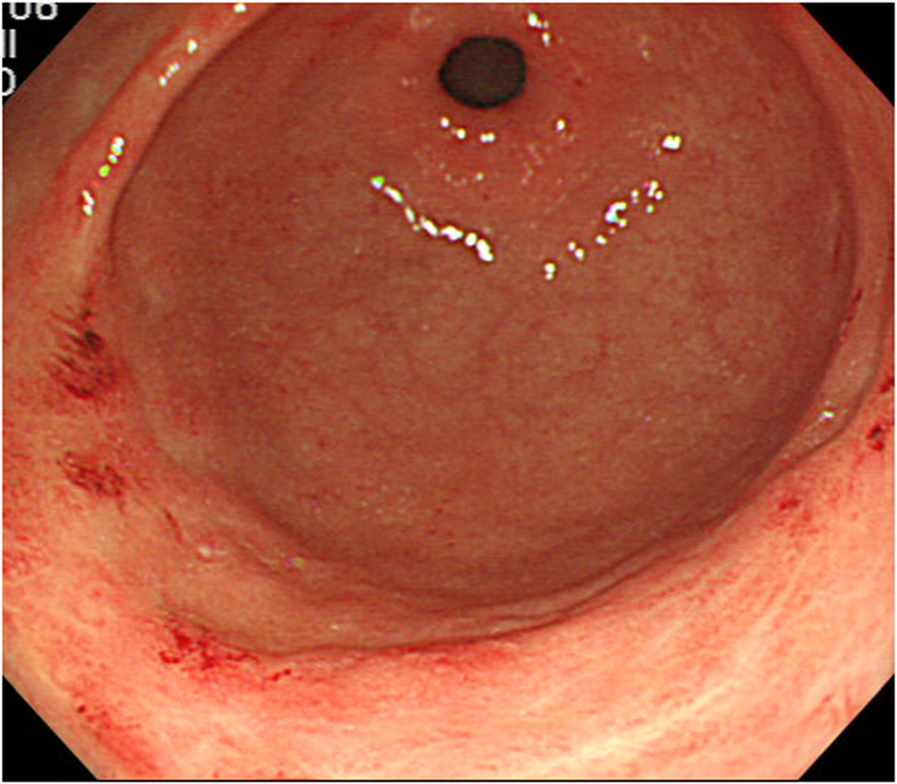
Esophagogastroduodenoscopy showed resolution of the patient’s gastric antral vascular ectasia 1 month after stopping temsirolimus, but diffuse angiodysplasia at the antrum was present.

Four weeks after discontinuing temsirolimus, the patient’s symptoms resolved and she was discharged in good clinical condition 60 days after hospital admission. One month after hospital discharge, significant improvement was seen in the gastric erythema and GAVE like lesions (Figure 3).

## Discussion

Six targeted agents for the treatment of advanced RCC are now approved and in clinical use: the tyrosine kinase inhibitors sunitinib and pazopanib, the multikinase inhibitor sorafenib, the anti–vascular endothelial growth factor monoclonal antibody bevacizumab, and the mammalian target of rapamycin (mTOR) inhibitors temsirolimus and everolimus [1].

Temsirolimus is an inhibitor of mTOR kinase, a component of intracellular signaling pathways involved in the growth and proliferation of cells [4,5] and the response of such cells to hypoxic stress [6]. The inhibition of angiogenesis by temsirolimus is clinically relevant because unregulated angiogenesis is prominent in RCC [7].

Minor hemorrhagic events are relatively common in patients treated with targeted agents; the most common event reported in patients treated with bevacizumab, sunitinib, temsirolimus, and everolimus is epistaxis, which usually resolves without medical attention. Life-threatening hemorrhagic events are rarer than minor hemorrhagic complications. In the case of bevacizumab, serious hemorrhage appears to be more frequently associated with specific tumor types, such as non–small cell lung cancer or cancer of the gastrointestinal tract [8].

GAVE, also known as watermelon stomach, is a rare cause of upper gastrointestinal bleeding that is often confused with portal hypertensive gastropathy, both of which can occur in patients with cirrhosis [9]. A clear relationship between mTOR inhibitors and the pathogenesis of targeted therapy-related stomatitis and GAVE has not been shown. In one case, a patient with gastrointestinal stromal tumors showed GAVE 8 months after starting imatinib [10]. Recently, a paper by Kim *et al*. [11] provided information on the activation of mTOR signaling pathways that promote wound healing in the stomach. Inhibitors of mTOR kinase may affect mucosal healing in the stomach and trigger gastritis and gastrointestinal bleeding.

Endoscopic therapy remains the treatment of choice for GAVE. Photocoagulation using a neodymium: yttrium-aluminum-garnet laser and APC have been successful in treating GAVE and abolishing or reducing transfusion requirements [12,13]. In the present case, several sessions of endoscopic hemostasis with APC effectively prevented severe anemia and blood loss due to gastrointestinal bleeding.

## Conclusion

Minor hemorrhagic events are relatively common in patients treated with targeted agents. Targeted therapy-related stomatitis and mucositis have occurred during targeted therapies, but there is no consensus on which strategy is the most effective. mTOR inhibitors may be related to the development of stomatitis, mucositis, and vascular ectasia through mTOR pathways. In the present case, endoscopic hemostasis with APC effectively prevented severe anemia and blood loss due to GAVE. This is the first case report of such an adverse drug reaction in the literature.

## Consent

Written informed consent was obtained from the patient for publication of this Case report and any accompanying images. A copy of the written consent is available for review by the Editor of this journal.

## Competing interests

The authors declare that they have no competing interests.

## Authors’ contributions

SF, HM, HK, TS, YH, MS and YK have made substantial contributions to acquisition of data and interpretation data. TM has given final approval of the version to be published. All authors read and approved the final manuscript.

## Pre-publication history

The pre-publication history for this paper can be accessed here:

http://www.biomedcentral.com/1471-2369/14/141/prepub

## Supplementary Material

Additional file 1 Endoscopic hemostasis with argon plasma coagulation (APC). Click here for file
